# Ilaprazole and Other Novel Prazole-Based Compounds That Bind Tsg101 Inhibit Viral Budding of Herpes Simplex Virus 1 and 2 and Human Immunodeficiency Virus from Cells

**DOI:** 10.1128/JVI.00190-21

**Published:** 2021-05-10

**Authors:** Jonathan Leis, Chi-Hao Luan, James E. Audia, Sara F. Dunne, Carissa M. Heath

**Affiliations:** a Department of Microbiology and Immunology, Northwestern University Feinberg School of Medicine, Chicago, Illinois, USA; b High Throughput Analysis Laboratory, Northwestern University, Evanston, Illinois, USA; c Department of Molecular Biosciences, Northwestern University, Evanston, Illinois, USA; d Visiting Scholar, Northwestern University, Evanston, Illinois, USA; e Chicago Biomedical Consortium, Northwestern University, Evanston, Illinois, USA; Lerner Research Institute, Cleveland Clinic

**Keywords:** budding of viruses, drugs to block budding, pan-antiviral agents

## Abstract

In many enveloped virus families, including human immunodeficiency virus (HIV) and herpes simplex virus (HSV), a crucial, yet unexploited, step in the viral life cycle is the release of particles from the infected cell membranes. This release process is mediated by host ESCRT complex proteins, which are recruited by viral structural proteins and provide the mechanical means for membrane scission and subsequent viral budding. The prazole drug tenatoprazole was previously shown to bind to the ESCRT complex member Tsg101 and to quantitatively block the release of infectious HIV-1 from cells in culture. In this report, we show that tenatoprazole and a related prazole drug, ilaprazole, effectively block infectious HSV-1/2 release from Vero cells in culture. By electron microscopy, we found that both prazole drugs block the transit of HSV particles through the cell nuclear membrane, resulting in their accumulation in the nucleus. Ilaprazole also quantitatively blocks the release of HIV-1 from 293T cells with a 50% effective concentration (EC_50_) of 0.8 to 1.2 μM, which is much more potent than tenatoprazole. Our results indicate that prazole-based compounds may represent a class of drugs with the potential to be broad-spectrum antiviral agents against multiple enveloped viruses by interrupting the cellular Tsg101 interaction with maturing virus, thus blocking the budding process that releases particles from the cell.

**IMPORTANCE** These results provide the basis for the development of drugs that target enveloped virus budding that can be used ultimately to control multiple virus infections in humans.

## Abstract (precis)

These results provide the basis for the development of drugs that target enveloped virus budding that can be used ultimately to control multiple virus infections in humans.

## INTRODUCTION

The advent of antibiotics had a major impact on controlling bacterial infections in patients worldwide, with a single drug being used to treat multiple infections. Unfortunately, antivirals have not had the same success. There are many factors contributing to this shortcoming, foremost the fact that few mechanisms are shared by different viruses, which limits targets for a broad-spectrum antiviral. Consequently, approved antivirals generally act against individual rather than groups of viruses, limiting a single drug’s potential.

Enveloped viruses bud from the host cell membranes and use the acquired lipid layer as a protective coat that also contains the glycoproteins required for infection of other cells. Enveloped viruses do not encode the machinery needed for budding and must recruit host cell proteins to bud from cells. In human immunodeficiency virus (HIV), ESCRT proteins are recruited to virus budding complexes through an interaction between the L domain (PT\SAP motif) in virus structural proteins (1–5, 67) and the cellular protein Tsg101 (tumor susceptibility gene 101), a homolog of the E2 ubiquitin (Ub)-conjugating enzyme and a member of the ESCRT-I complex (4, 6–9). Tsg101 recruits the cellular ESCRT-III complex, which provides the mechanical means for viruses to passage through cell membranes to be released from cells (8, 10–17). In contrast to HIV, herpes simplex virus (HSV) assembles particles in the nucleus and must passage through the nuclear membrane into the cytoplasm, where it exchanges membranes to become infectious and is then released from the cell membrane. The ESCRT proteins are required for this passage (18–20). Thus, virus budding may present a common target for treating multiple virus infections.

In support of targeting this pathway, a recent seminal discovery in our laboratory established that an interferon-induced protein, interferon-stimulated gene 15 (ISG15), specifically targets the ESCRT-III proteins in budding complexes to block the release of viruses (21–24). This indicates that the human immune system evolved to target the ESCRT pathway to control viral infections and supports that this is a natural target. Another group identified single-nucleotide polymorphic sites in the 5′ region of Tsg101, located at positions −183 and +181 relative to the translation start signal, which affect the rate of AIDS progression among Caucasians (25). These data support the hypothesis that variation in Tsg101 affects the efficiency of Tsg101-mediated release of viral particles from infected cells, altering plasma viral load levels and subsequent disease progression. Taken together, these investigations indicate that Tsg101 and ESCRT proteins present a natural antiviral target.

Currently, the prazole family of drugs is best known for their role as proton pump inhibitors (PPIs), and a few, namely, omeprazole (Prilosec), esomeprazole (Nexium), and ilaprazole (Adiza, Noltec, and Yi Li An), are marketed to control symptoms of gastroesophageal reflux disease (GERD) in either the United States or abroad. PPIs form a covalent bond with the active site of proton pumps, inhibiting their ability to acidify the stomach and reducing symptoms associated with overacidification (26). Recent reports indicate that drugs from the prazole family, including tenatoprazole and esomeprazole, form a disulfide linkage to Tsg101, which results in blocking the release of HIV-1 from cells in culture (3).

In the present manuscript, we demonstrate that multiple prazole drugs block the budding of HSV-1 and HSV-2 from Vero cells in culture, strengthening the case for the broad-spectrum potential of this mechanism/drugs. Most notably, we identified one prazole drug, ilaprazole, that blocks the release of both HIV-1 and HSV-1/2 from cells with an efficiency more potent than that reported for tenatoprazole. Ilaprazole acts in the low-micromolar range without detectable cell toxicity at inhibitory concentrations. To further define the mechanism of action of these prazole drugs on HSV infections, we identified the site of blockage of herpesvirus release, which appears to be different from that of HIV-1. While the blockage of HIV-1 particle release is at the outer cell membrane (3), the prazole drugs appear to first block the passage of herpesvirus through the nuclear membrane. This prevents particles from being released into the cytosol, where maturation of their envelope membrane occurs to produce infectious virus and where they bud from the cell. With the prazole-based inhibitors being effective in both HIV and HSV, targeting Tsg101 could lead to a broad-spectrum antiviral therapy.

## RESULTS

### Identification of prazole compounds that bind the UEV ubiquitin-binding domain of Tsg101.

**FIG 1:**
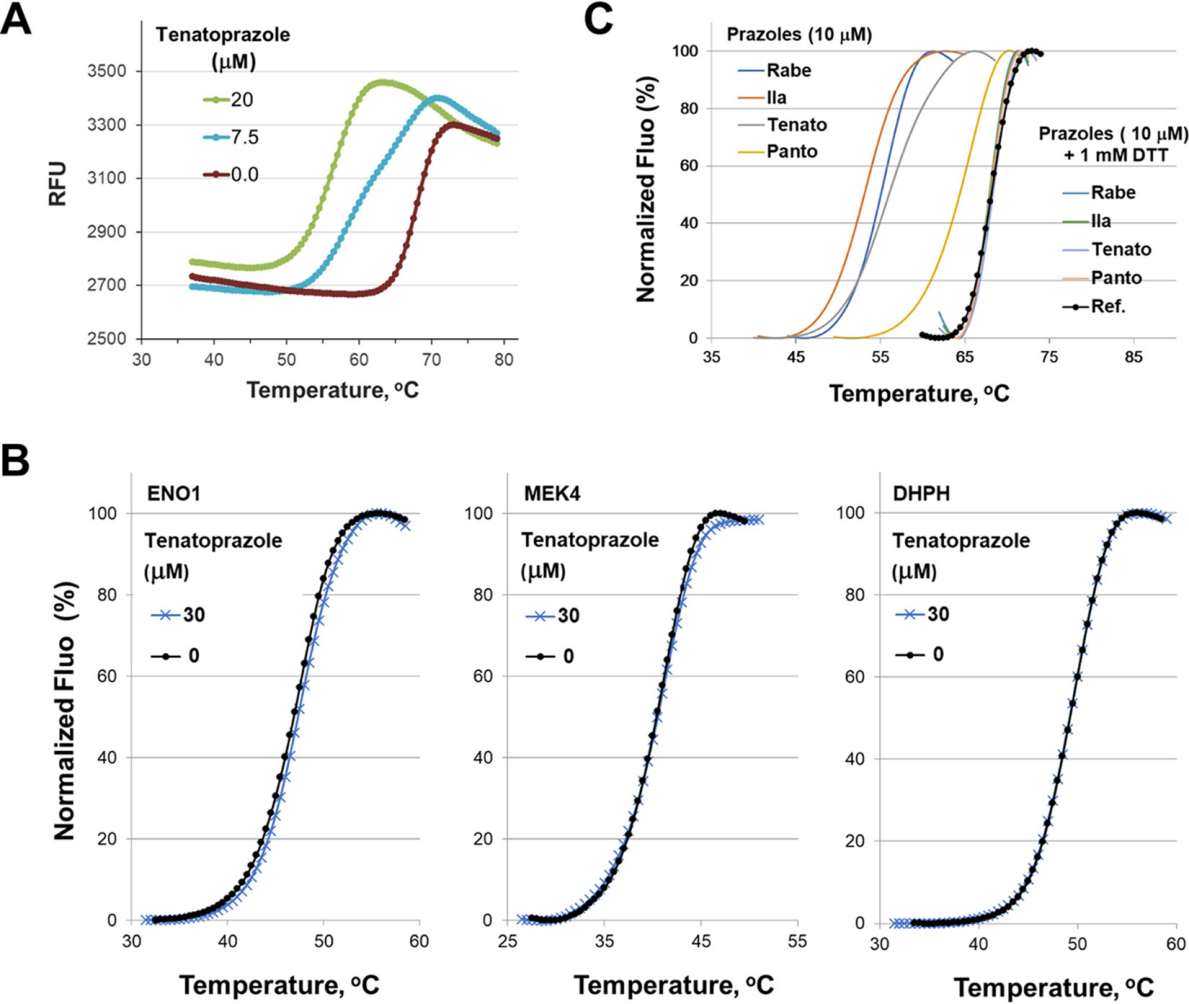
Thermal shift data of Tsg101 by the lead compound tenatoprazole (N16). (A) The compound caused a dose-dependent shift in the *T_m_* for Tsg101-UEV, indicating binding to the key domain of Tsg101, as described in Materials and Methods. RFU, relative fluorescence units. (B) DHPH, ENO1, and MEK4 do not cause a thermal shift with Tsg101. Thermal shift data of three human proteins not related to Tsg101 by the lead compound tenatoprazole are shown. The effect of the prazole compound on the thermal stability of these three proteins is negligible, indicating that the dramatic modulation of the thermal transition of Tsg101 by the prazoles is due to a specific interaction. (C) The addition of DTT abolishes the *T_m_* shift in the FTS assay, consistent with prazole compounds forming a disulfide bond to Tsg101. Rabe, rabeprazole; Ila, ilaprazole; Tenato, tenatoprazole; Panto, pantoprazole; Ref., reference; Fluo, fluorescence.

We screened chemical compounds using a fluorescence thermal shift (FTS) assay (27, 28) to identify small molecules that bind directly to a truncated form of Tsg101 (amino acids [aa] 1 to 145), which contains the ubiquitin E2 variant (UEV) ubiquitin-binding domain (Fig. 1). The UEV, which contains the PT/SAP-binding domain in addition to the ubiquitin-binding domain, provides chaperone functions to HIV-1 Gag, which is independent of its interaction with the PS/TAP motif, and contains the prazole-binding site (3). This truncated Tsg101, called Tsg101-UEV, was used because full-length Tsg101 has significant solubility issues in aqueous solution. Tsg101 is an adaptor protein and thus lacks a readily deployable functional assay, making the FTS assay a tractable approach to identify interacting compounds. FTS monitors protein thermal denaturation using SYPRO orange, a dye that fluoresces when bound to hydrophobic surfaces, which allows monitoring of the changes in hydrophobic surface exposure during protein denaturation (27). Since ligand binding affects protein thermal stability, it can be detected through modulation of protein thermal denaturation (melting) as a shift in the melting temperature (*T_m_*). Tsg101-UEV has a well-defined melting curve suitable for FTS. We used the FTS assay to identify compounds that bind to Tsg101-UEV.

We compared the thermal denaturation profiles for Tsg101-UEV in the presence and absence of tenatoprazole and found that it destabilizes the native protein structure, indicating that it binds Tsg101-UEV (Fig. 1A). We also tested tenatoprazole against proteins unrelated to Tsg101, including DHPH, ENO1, and MEK4, and did not observe a *T_m_* shift, indicating that the *T_m_* shift of Tsg101-UEV was due to a specific interaction of the prazole compound (Fig. 1B). This specific binding is consistent with a previous nuclear magnetic resonance (NMR) structure in which tenatoprazole forms a covalent disulfide bond to Cys73 in the UEV domain of the protein (3). This disulfide bond formation can be prevented by including the reducing agent dithiothreitol (DTT) in the assay (Fig. 1C). The addition of DTT abolished the Tsg101-UEV *T_m_* shift caused by the prazoles. Therefore, the addition of DTT to the FTS assay is a facile means to ascertain if prazole analogs interact with Tsg101-UEV in a covalent manner.

### Tenatoprazole inhibits herpesvirus release from Vero cells.

Tenatoprazole and esomeprazole were shown to quantitatively inhibit the release of infectious HIV-1 from 293T cells in culture, and it was suggested that these effects may be mediated via changes in the viral interaction with Tsg101, a key component of the cellular ESCRT complex (3, 20). Given multiple reports suggesting that herpesviruses also use cellular ESCRT proteins in their replication process (18, 19), we tested if the Tsg101-binding prazole drugs, which blocked the budding of HIV-1, would also block the release of herpesviruses from cells.

We infected Vero cells with HSV-1 and HSV-2 for 2 h at a multiplicity of infection (MOI) (in PFU per cell) of 0.1 to assay the antiviral activity of tenatoprazole. Following infection, cells were treated with different concentrations of tenatoprazole. After 24 or 48 h, the medium fractions were collected, and released virus titers were determined by standard plaque assays (29). Tenatoprazole caused a 3-log drop of HSV-1 and a 4- to 5-log drop of HSV-2 in infectious virus released from Vero cells 24 h after infection in a dose-dependent manner (Table 1, 2nd and 3rd columns), with calculated 50% effective concentrations (EC_50_s) ranging from 48 to 80 μM. Similar results were obtained 48 h after infection (Table 1, 5th and 6th columns). The total virus titer was also determined to differentiate between virus released into the medium and infectious particles present in the cell lysate. Total infectious virus particles were reduced by tenatoprazole but not as strongly as virus released into the medium (Table 1, compare the 3rd and 4th columns). The concentrations of tenatoprazole that blocked virus release were nontoxic to Vero cells as determined by a 96 AQueous One Solution cell proliferation assay reagent (Table 1, 7th column). Taken together, the results show that tenatoprazole inhibited the levels of both released and infectious virus particles without affecting cell viability at effective concentrations.

**TABLE 1:** Effect of tenatoprazole on HSV-1 and-HSV-2 release from Vero cells^*a*^

Tenatoprazole concn (μM)	Titer of HSV-1 in medium at 24 h	Titer of HSV-2 in medium at 24 h	Total titer of HSV-2 in medium + cell lysate at 24 h	Titer of HSV-1 in medium at 48 h	Titer of HSV-2 in medium at 48 h	Viability (OD_490_) at 24 h
0	2.50E+05	2.80E+05	4.70E+07	8.0E+07	8.50E+06	1.694
52	2.90E+05	6.50E+04	4.00E+06	1.4E+07	1.30E+06	1.724
60	ND	1.00E+03	2.30E+04	ND	5.60E+04	1.759
79	1.30E+05	2.50E+02	1.50E+03	8.0E+06	4.80E+03	1.742
105	5.40E+04	0.00E+00	6.00E+02	5.3E+05	1.80E+02	1.777
131	2.40E+03	0.00E+00	3.00E+02	3.5E+04	ND	1.714
157	1.30E+02	ND	ND	1.0E+02	ND	ND
200	ND	ND	ND	ND	ND	0.872

a Tenatoprazole was incubated with Vero cells infected with HSV-1 or HSV-2 at a range of concentrations. The amount of virus released into the medium fraction at the indicated times was determined as described in Materials and Methods. Total virus is the amount of virus released from cells plus the amount of virus inside the cells. The viability of Vero cells incubated with increased concentrations of tenatoprazole was determined using the 96 AQueous One Solution cell proliferation assay reagent as described in Materials and Methods. The total titer for HSV-1 was not included. Duplicate plaque assays of 10-fold serial dilutions were performed, with an average of a <13% difference in the number of plaques counted. The 24-h and 48-h assays were repeated 6 times each. The total virus assay was repeated twice. The data presented are the averages from 2 experiments where the titers varied between 5 and 20%. OD_490_, optical density at 490 nm; ND, not determined.

### Cellular location of tenatoprazole inhibition.

**FIG 2:**
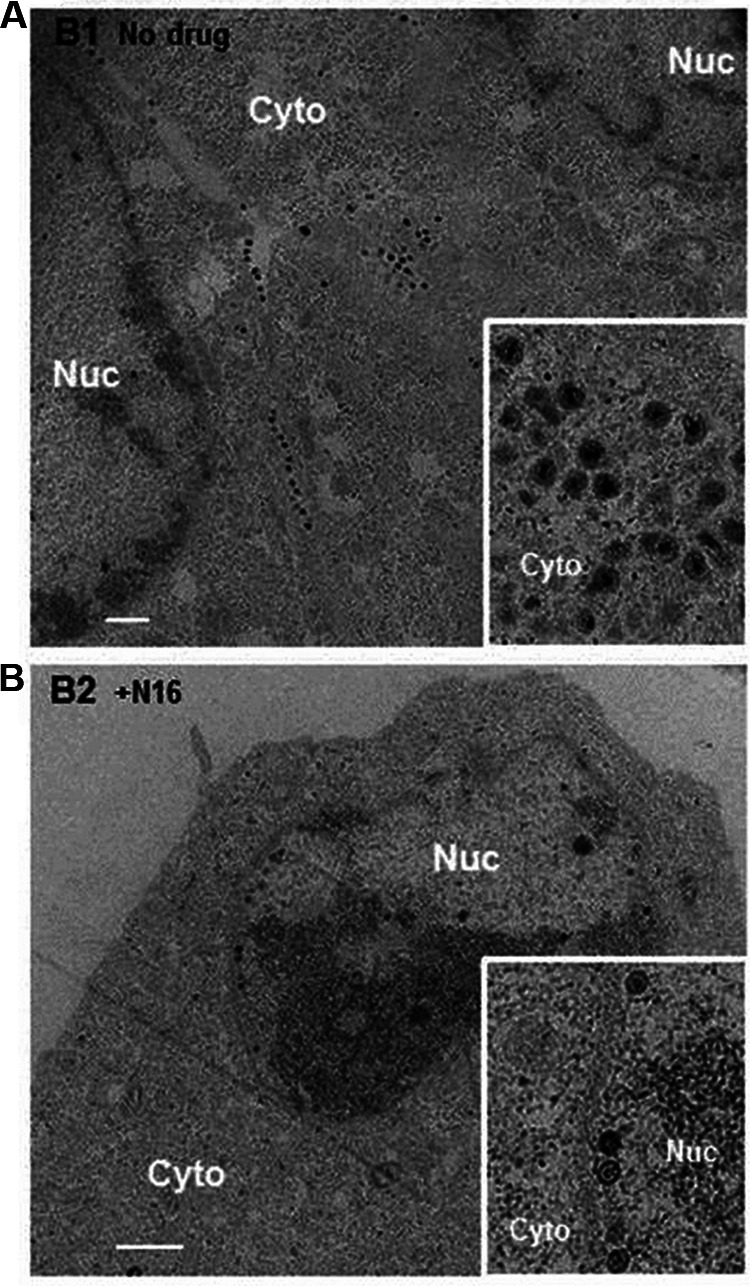
Inhibitory effect of tenatoprazole on HSV-2 production and location of virus particles inside infected cells. Cells with virus were untreated (A) or treated with 105 μM tenatoprazole (N16) (B) for 24 h and examined by transmission electron microscopy. In each case, 80 cells where virus particles were observed were examined. For untreated cells, we observed an average of 120 particles in the cytoplasm (Cyto), ranging from 28 to 204 particles. In the nucleus (Nuc), we observed an average of 16 particles, ranging from 10 to 22 particles. In the presence of the drug, we observed a significant increase in dense material in the nucleus with some particles associated with it. We observed an average of 31 particles, ranging from 8 to 48 particles. In the cytoplasm, we did not detect virus particles. Bar = 1 μm. The inset shows a higher-magnification image.

We next imaged herpesvirus-infected Vero cells using transmission electron microscopy (TEM) to determine the site of inhibition of release of virus and whether it was similar to observations of HIV-1 release from 293T cells. Vero cells grown on glass coverslips were infected with HSV-2 at an MOI of 0.1 PFU/cell for 2 h and then treated for 24 h with 105 μM tenatoprazole or the vehicle control. Using electron microscopy, we examined 80 cells with virus particles, and representative images are shown in Fig. 2. In the no-drug control, virus particles were in both the nucleus and the cytoplasm near the cell surface (Fig. 2A). In the tenatoprazole-treated cells, the cytosol of all of the intact cells was largely devoid of virus particles (Fig. 2B). Instead, we observed large pockets of granular material accumulated in the nucleus and immature virus particles inside the nucleus and lining the inside of the nuclear membrane (Fig. 2B, inset). This result suggests that tenatoprazole blocks the passage of herpesvirus particles through the nuclear membrane, in contrast to a report by Pawliczek and Crump (30). This result also differs from that observed for HIV-1. Because tenatoprazole binds Tsg101, this suggests that the ESCRT-I protein complex is involved in the transport of HSV-2 through the nuclear membrane and/or particle assembly.

### Identification of potent prazole-based inhibitors.

Despite the lack of a cell toxicity signal at effective tenatoprazole concentrations, the effective concentration is too high for use as clinical therapy. Therefore, more potent analogs are required to explore the antiviral therapeutic potential. We set out to identify and test other analogs that were more potent prazole analogs. We began by searching PubChem for analogs of tenatoprazole. We identified and obtained a dozen such compounds from commercial sources and prioritized these for testing based on structural similarities around the sites where tenatoprazole covalently linked to Cys73 of Tsg101. To this end, tenatoprazole, lansoprazole, rabeprazole, dexlansoprazole, pantoprazole, esomeprazole, 4-desmethoxy-omeprazole (an omeprazole analog, 5-methoxy-2{[(3,5-dimethyl-4-methoxy-pridin-2-yl-*N*-oxide)methyl]sulfinyl}-1H-benzimidazole; labeled O-omeprazole), omeprazole, and ilaprazole were assessed in the FTS assay for their ability to change the *T_m_* of Tsg101-UEV as described above (data not shown).

**FIG 3:**
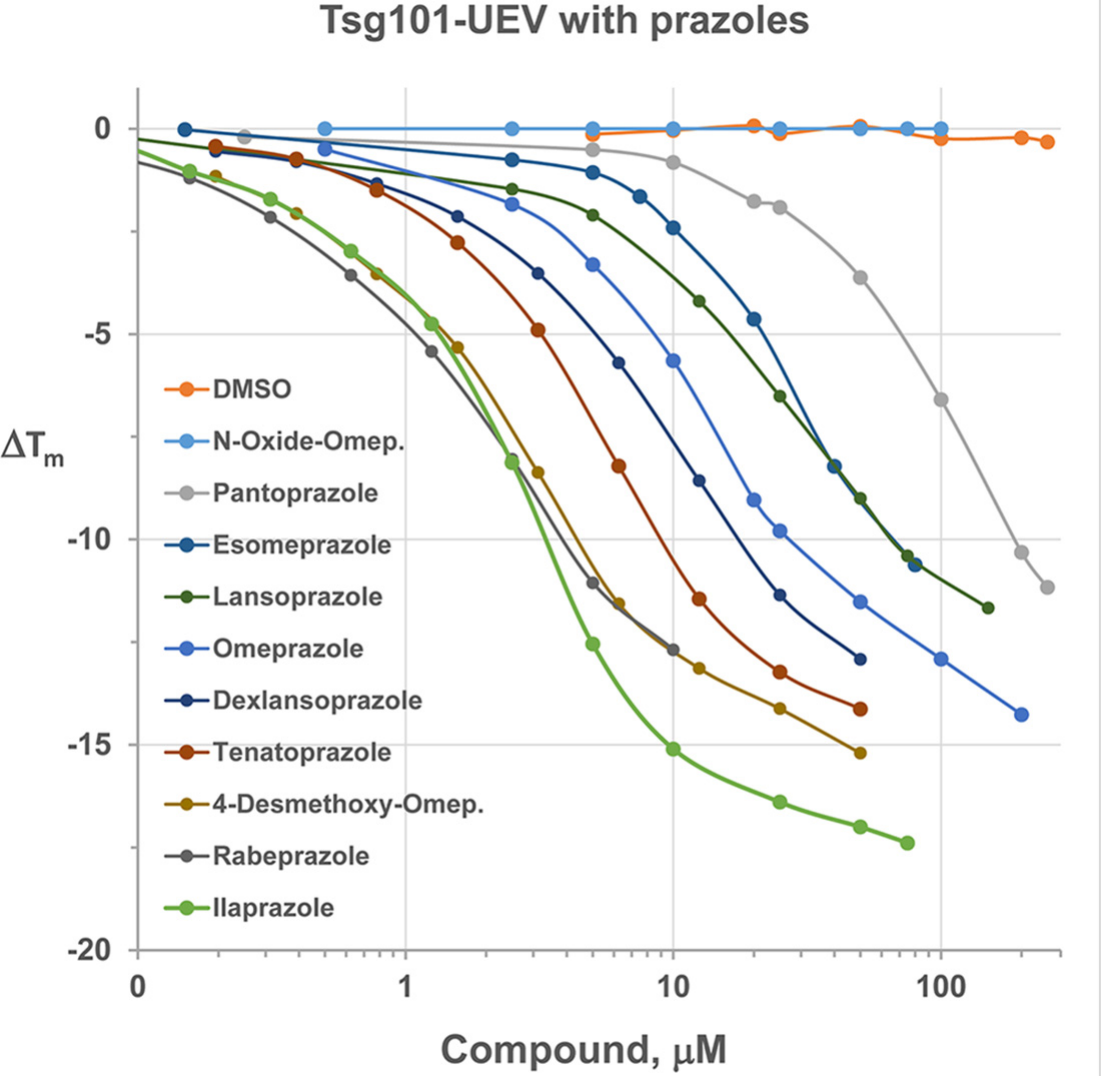
Dose-response plots of Tsg101 melting temperature shifts caused by 10 prazole compounds. Different concentrations of prazole compounds were incubated with Tsg101 (aa 1 to 145) and subjected to fluorescence thermal shift analysis as described in Materials and Methods.

We determined the dose-response plots of Tsg101 melting temperature shifts caused by these prazole compounds binding to Tsg101(1–145) (Fig. 3). O-omeprazole is the only compound predicted not to form a covalent bond with Tsg101 since it has an oxygen linked to a ring nitrogen that is normally a hydrogen in the active prazoles (Table 2, right column). As expected, O-omeprazole did not cause a detectable thermal shift (Fig. 3). The smallest thermal shift was observed with pantoprazole (Fig. 3, gray), and the largest thermal shift was observed with ilaprazole (green). Ilaprazole’s ability to cause a thermal shift with Tsg101 was blocked by the addition of DTT (Fig. 1C), consistent with the idea that the compound forms a disulfide linkage to Tsg101.

**TABLE 2:**
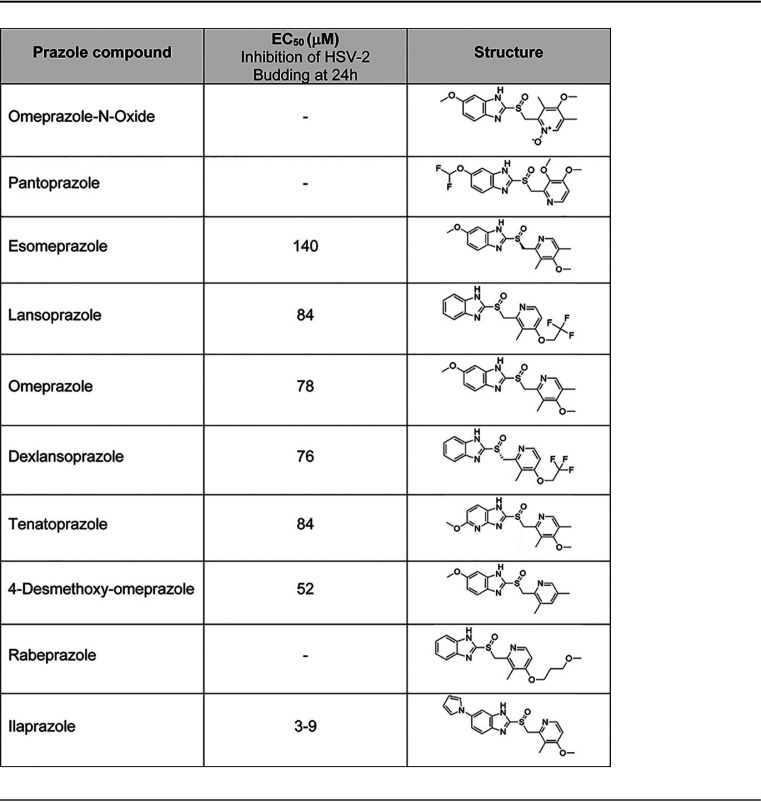
Effect of commercial prazole analogs to inhibit the release of HSV-2 from Vero cells^*a*^

a Different concentrations of the listed prazole drugs were incubated with HSV-2-infected Vero cells for 24 h, and virus released into the medium was then quantified by plaque assays. Data presented include the EC_50_ value (concentration at which virus release is inhibited by 50%). Methods are described in the footnote of Table 1.

Next, we tested the antiherpesvirus activity of these prazole compounds (Table 2). To examine the effects of the compounds on the release of HSV-2 from Vero cells, we infected the cells with virus 2 h prior to treatment with medium containing different concentrations of the compound. We incubated the cells for 24 or 48 h and then collected the cell medium fractions. Several of the analogs were inactive, including O-omeprazole, pantoprazole, and rabeprazole. We identified a number of active compounds, in which there was a 10-fold spread of inhibition activity against HSV-2, ranging from an EC_50_ of 140 μM (for esomeprazole) to an EC_50_ of 3 to 9 μM (for ilaprazole). Thus, we identified prazole analogs that are more potent than tenatoprazole.

We provide the structures of prazole compounds tested in this analysis (Table 2, 3rd column). Of note, ilaprazole contains an additional ring structure compared to tenatoprazole that is predicted to lie in a solvent-exposed area of the Tsg101 structure that may serve to strengthen the interaction with Tsg101. In examining the thermal shift capacity of the prazoles, we found that the larger the thermal shift, the more potent the antiviral activity associated with the compound. This correlation indicates that the FTS assay is useful in evaluating structure-activity relationships (SARs) to inform the design of new compounds (Fig. 3 and Table 2).

### Antiviral activity of ilaprazole on HSV-1 and HSV-2 *in vitro*.

Based on these initial HSV-2 antiviral assay results, we selected ilaprazole for further antiviral profiling and tested it against HSV-1 (Table 3, 2nd to 5th columns) and HSV-2 (Table 3, 6th to 8th columns). Ilaprazole was slightly more effective against HSV-1 than against HSV-2, with EC_50_ calculations ranging from 3 to 9 μM. These results do not indicate if the observed lower EC_50_ against HSV-1 than against HSV-2 is significant or reflects differences between different viral isolates since the two herpesviruses can be distinguished by sequence analysis, and both types can cause oral and genital lesions. Ilaprazole’s potency is an improvement over tenatoprazole, which inhibited in the high-micromolar range (Tables 1, 3, and 4). Like tenatoprazole, ilaprazole caused a significant drop in total virus, again not as strong of a decrease as that one detected with virus released from cells. Additionally, ilaprazole was even more effective in inhibiting virus release at 72 h than at 24 h after a single application of the drug (72-h EC_50_, 0.8 to 1.2 μM) (Table 3, compare the 2nd and 4th columns). Significant inhibition was still observed 4 and 5 days after a single application of the drug (data not shown). The inhibition caused by tenatoprazole against either virus began to fall off after 48 h (data not shown). We also tested for toxicity in the range of effective concentrations and did not observe cell toxicity using the 96 AQueous One Solution cell proliferation assay reagent and the WST-1 reagent over a 24-h period (Table 4). Thus, ilaprazole is more potent and has longer-lasting effects than tenatoprazole.

**TABLE 3:** Effect of ilaprazole on the release of HSV-1 and HSV-2 from Vero cells^*a*^

Ilaprazole concn (μM)	Titer of HSV-1 in medium at 24 h	Titer of HSV-1 in medium at 48 h	Titer of HSV-1 in medium at 72 h	Total HSV-1 titer in medium + cell lysate at 24 h	Titer of HSV-2 in medium at 24 h	Total HSV-2 titer in medium + cell lysate at 24 h	Titer of HSV-2 in medium at 48 h
0	3.00E+06	3.90E+07	1.00E+08	2.2E+08	2.80E+05	1.20E+07	1.00E+06
4.5	2.00E+06	2.40E+06	9.00E+05	7.0E+07	1.00E+05	3.60E+07	2.50E+05
9.0	7.50E+04	2.50E+05	2.20E+05	3.6E+06	5.00E+04	4.50E+06	7.50E+04
13.5	3.20E+04	2.00E+02	4.50E+02	3.8E+05	1.00E+04	4.30E+06	5.50E+04
18.0	6.00E+02	0.00E+00	1.00E+02	9.1E+03	1.50E+03	2.00E+05	1.50E+03
22.5	1.00E+02	0.00E+00	ND	7E+02	1.00E+02	1.50E+04	3.00E+02
54	ND	ND	ND	ND	ND	ND	ND
270	ND	ND	ND	ND	ND	ND	ND

a Different concentrations of ilaprazole were incubated for the times indicated with HSV-1- or HSV-2-infected cells, similar to the methods described in the footnote to Table 1. Titers of virus released into the medium and total virus were determined. Data were analyzed as described in the footnote to Table 1, and experiments were repeated 4 times each.

**TABLE 4:** Effect of ilaprazole on the viability of HSV-1- and HSV-2-infected Vero cells^*a*^

Ilaprazole concn (μM)	96 AQueous One Solution cell viability (OD_490_) at 24 h	WST-1 cell toxicity (OD_440–660_) at 24 h
0	1.694	0.993
4.5	1.764	1.058
9.0	1.711	1.055
13.5	1.690	0.950
18.0	1.737	ND
27	ND	1.055
54	1.658	ND
270	0.466	0.423

a The viability of Vero cells incubated with increasing concentrations of ilaprazole was determined using the 96 AQueous One Solution cell proliferation assay reagent as described in Materials and Methods. Data were analyzed as described in the footnote of Table 1, and experiments were repeated 4 times each.

**FIG 4:**
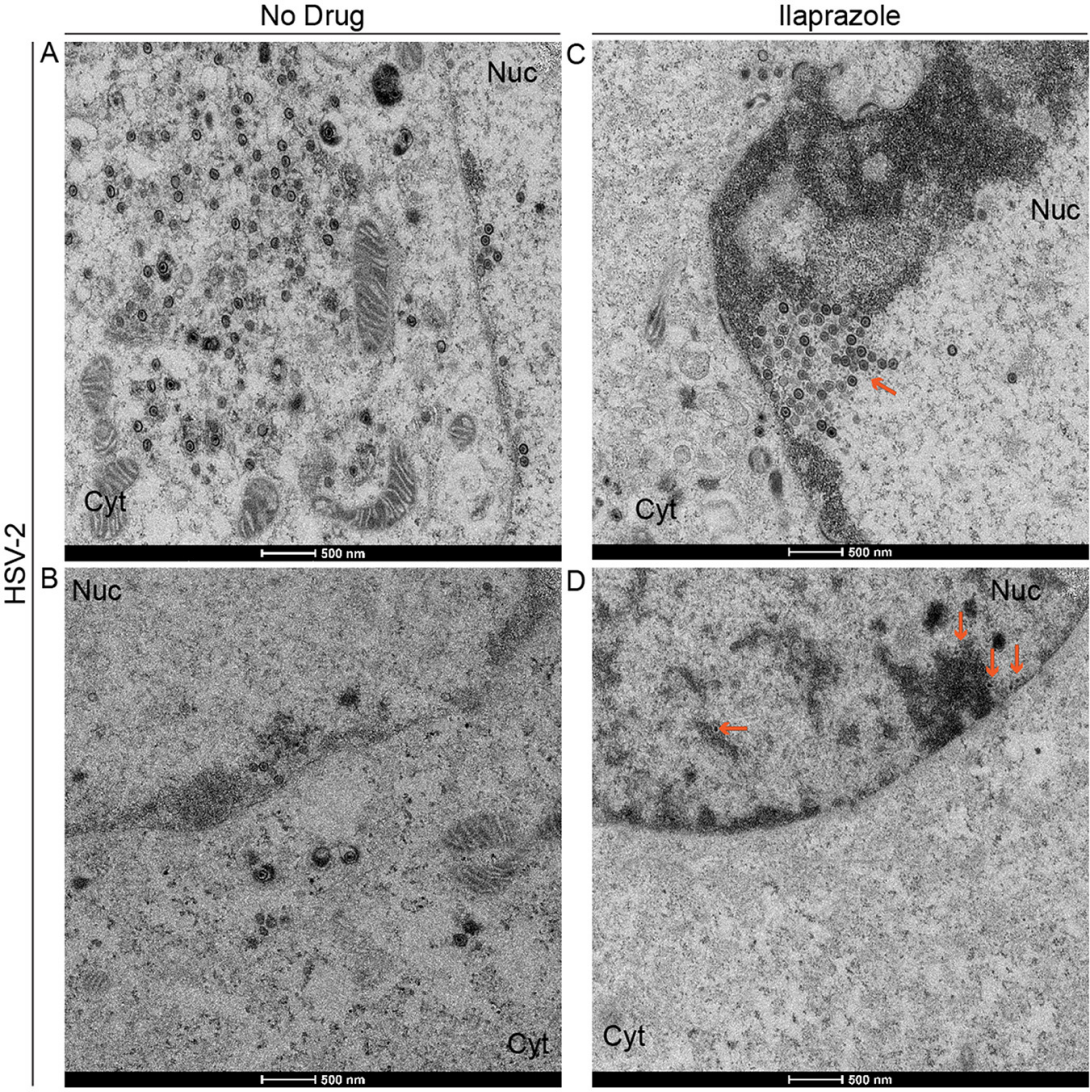

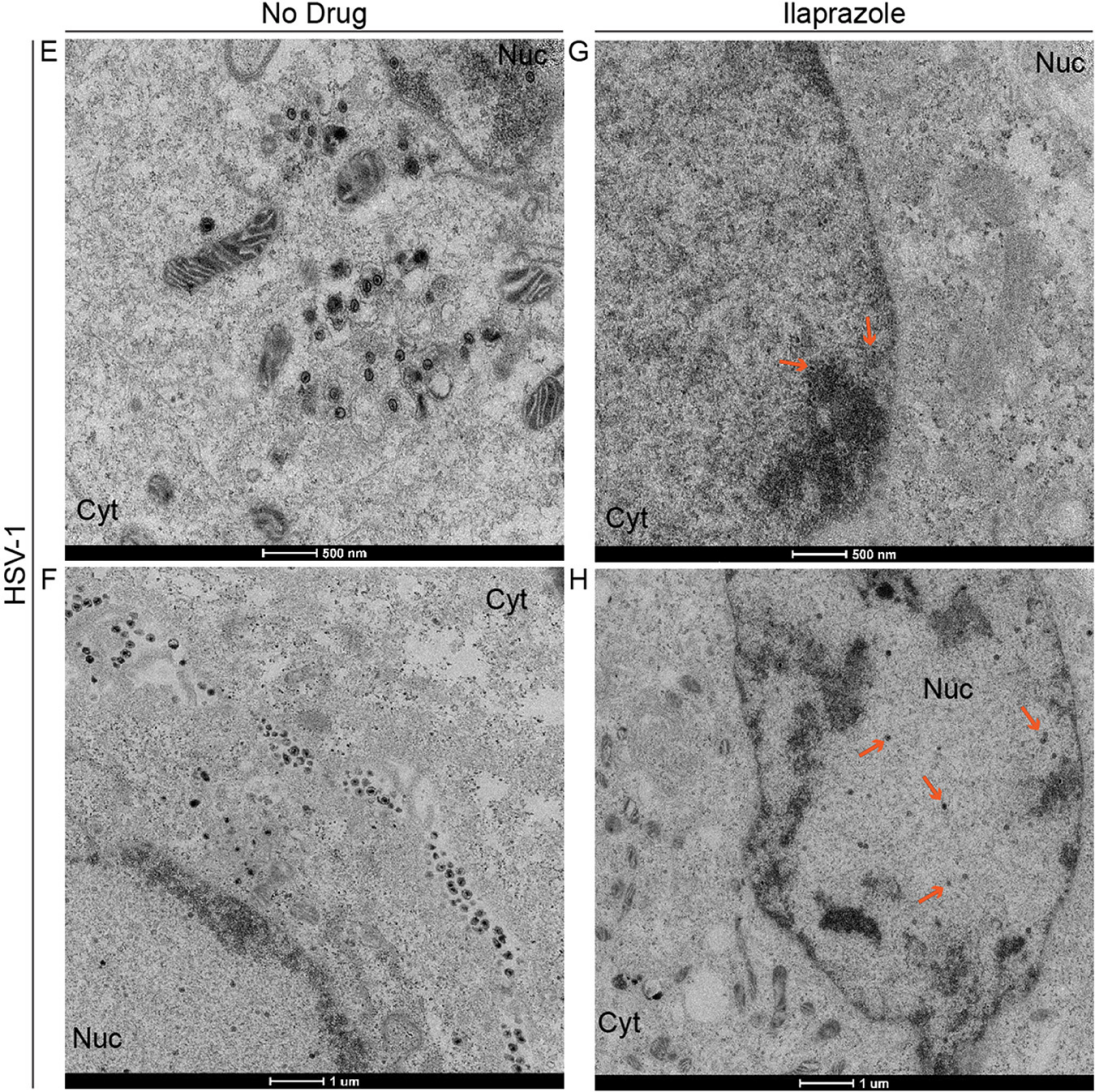
Inhibitory effect of ilaprazole on HSV-2 and HSV-1 production. Vero cells were infected with HSV-2 or HSV-1 at an MOI of 0.1 and examined by transmission electron microscopy 24 h later. (A to D) HSV-2-infected untreated cells (A and B) and cells treated with 18 μM ilaprazole (C and D). (E to H) HSV-1-infected untreated cells (E and F) and cells treated with 18 μM ilaprazole (G and H). Eighty cells where virus particles were observed were examined. In the presence of ilaprazole, we observe no or very few particles in the cytoplasm (Cyt). Treated cells also have an accumulation of electron-dense material and particles associated with them. In untreated HSV-2-infected cells, we observed an average of 120 particles in the cytoplasm, ranging from 28 to 204, and an average of 16 particles, ranging from 10 to 22, in the nucleus. In treated HSV-2-infected cells, we observed 0 particles in the cytoplasm and an average of 31 particles, ranging from 9 to 56 particles, in the nucleus. In untreated HSV-1-infected cells, we observed an average of 135 particles, ranging from 12 to 152 particles, in the cytoplasm and an average of 35 particles, ranging from 10 to 66 particles, in the nucleus. In treated HSV-1-infected cells, we observed 0 particles in the cytoplasm and an average of 43 particles, ranging from 15 to 166 particles, in the nucleus. Arrows point to virus particles.

We next carried out a transmission electron microscopic examination of cells infected with HSV-2 at an MOI of 0.1 in the presence and absence of 18 μM ilaprazole to determine if this drug causes the accumulation of virus particles in the nucleus of cells similar to that caused by tenatoprazole. Without the drug, we observe particles in the cytoplasm and the nucleus (Fig. 4A and B); in the presence of the drug, few or no viral particles are found in the cytoplasm (Fig. 4C and D). In both heavily infected cells (Fig. 4A and C) and mildly infected cells (Fig. 4B and D), treatment led to particles being detected in the nucleus and arrayed along the nuclear membrane but lacking in the cytosol. This indicates that the location of particles in the cell in the presence of the drug is independent of the number of particles observed. Similar results were obtained with HSV-1-infected cells (Fig. 4E to H). Particles are seen in both the cytoplasm and nucleus in the absence of the drug and just in the nucleus in the presence of the drug. These results are similar to the effect of tenatoprazole on HSV-2-infected cells (Fig. 2). The lower total infectious virus titers shown in Table 3 are consistent with a blockage of the virus passaging out of the nucleus into the cytoplasm where membranes are exchanged and virus becomes infectious.

### Effect of ilaprazole on the release of HIV-1 *in vitro*.

To establish the broad-spectrum potential of ilaprazole, we tested whether ilaprazole would inhibit the release of HIV-1 from 293T cells. To this end, cells were transfected with the pR9-HIV-1_Ba-L_ plasmid, and the release of virus into the medium fraction was detected by monitoring the capsid (CA) protein (p24) via an enzyme-linked immunosorbent assay. The drug was tested at concentrations of between 0 and 40 μM, and the effect of the drug on the release of virus was assessed (Table 5, 2nd column). Ilaprazole was effective at inhibiting the release of HIV-1 from cells, with a calculated EC_50_ of 1 μM or lower, as described in Materials and Methods. We did not detect toxicity to the cells at the drug concentrations that inhibited the release of HIV-1 over the course of these experiments. Thus, ilaprazole has antiviral activity against HSV-1, HSV-2, and HIV-1, demonstrating its potential as a broad-spectrum antiviral.

**TABLE 5:** Effect of ilaprazole and novel analogs on the release of HIV from 293T cells^*a*^

Ilaprazole concn for virus titer determination (μM)	HIV p24 concn (pg/ml)	Ilaprazole concn for cell viability determination (μM)	96 AQueous One Solution cell viability (OD_490_) at 24 h
0	7,021	0	1.694
0.5	4,063	1.8	1.672
1	3,518	3.6	1.576
2	1,652	7.2	1.684
5	274	10.8	1.650
10	178	13.5	1.693

a Different concentrations of ilaprazole were incubated with HIV-1 plasmid-transfected cells as described in Materials and Methods. The titer of virus released into the medium was determined by monitoring p24 levels at 24 h postinfection using a fluorescence-labeled antibody. Each experiment was repeated 4 times, and the average p24 CA concentrations in picograms per milliliter are presented. Cell toxicity experiments were repeated 2 times each.

## DISCUSSION

We are developing a novel strategy to treat viral infections affecting humans by disrupting a common mechanism used by many enveloped viruses to bud from cells. Viral budding inhibitors (VBIs) have the potential to be broad-spectrum antiviral therapeutics, potentially being effective against herpesvirus (20, 30–34), retro/lentiviruses (3, 20), arenaviruses (Lassa fever virus [LFV] and lymphocytic choriomeningitis virus [LCMV]) (35, 36), flaviviruses (hepatitis C virus [HCV]) (37, 38), filoviruses (Ebola virus and Marburg virus [MarV]) (39–46), hepadnaviruses (hepatitis B virus [HBV]) (47), some paramyxoviruses (simian virus 5 [SV5] and mumps virus [MuV]) (48–50), and rhabdoviruses (vesicular stomatitis virus [VSV] and rabies virus [RV]) (7, 51, 52). VBIs would require testing for antiviral activity against these different viruses before clinical use but nonetheless present a strong starting point for identifying therapeutics.

In this work, we demonstrate the antiviral activity of prazole compounds, with no detectable cell toxicity at effective concentrations, against two viruses that use different mechanisms for viral replication. Of particular note is that the viral genomes are very different, with HIV being RNA based and HSV being DNA based. That one compound works against viruses with such stark differences in viral life cycle types supports that these compounds have potential as broad-spectrum antiviral agents for current and emerging viruses. This aspect gives this approach an advantage over other potential broad-spectrum antivirals such as remdesivir, which is targeted to RNA viruses, limiting its potential as a broad-spectrum antiviral (53).

Tsg101 binding to the proline-rich viral L domains in Gag (1, 4, 5, 9, 12, 13) is required for virus particles to be released from cell membranes of infected cells. Tsg101 is a member of the ESCRT-I complex of proteins involved in cell endosomal sorting. The ESCRT-I complex recruits proteins from the ESCRT-III complex with AIP1 (17), which provides the mechanical means for the scission of virus particles from cell membranes. Thus, blocking the PT/SAP L-domain sequence from interacting with the host ESCRT complex causes the virus budding defect, and three lines of independent evidence support this idea. First, drugs targeted to this specific interaction in HIV-1 cause virus budding defects in infected cells without detectable off-target effects (3). Second, a research group identified noncoding single-nucleotide polymorphisms (SNPs) in the 5′ region of Tsg101 that correlate with viral loads, implicating Tsg101-mediated viral particle release in disease progression (25). Third, viral infections activate a host innate immunity mechanism, through interferon-stimulated gene 15 (ISG15), that specifically disrupts virus budding complexes (21). In response to this immune system defense, many viruses encode enzymes that prevent or reverse ISG15 conjugation to cellular proteins to avoid the budding blockade (54–59). Taken together, this evidence indicates that targeting this interaction may lead to an effective antiviral strategy. Note that Pawliczek and Crump (30) have reported that HSV-1 production requires a functional ESCRT-III complex that could be independent of Tsg101 and Alix expression. However, there are multiple pathways to recruit ESCRT-III proteins to functional virus budding complexes. For example, if we genetically replace the PT/SAP with the PPPPY L domain in HIV-1 Gag, the virus still buds from cells independent of Tsg101 (8). Also, mutations of the HIV-1 L domain in Gag cause a budding defect that can be rescued by the overexpression of the specific ubiquitin ligase Nedd4L (10, 60). Nedd4L normally binds PPPPY motifs, which are absent from HIV-1 Gag. However, Nedd4 interacts with ESCRT-II proteins downstream from Tsg101, which in turn recruits the ESCRT-III proteins to the virus budding site (Leis and Seo, unpublished data). Thus, while Tsg101 is normally involved in recruiting the ESCRT-III complex, under stress, its function can be replaced. This motivates our parallel investigation of small-molecule inhibitors that target Nedd4’s recruitment of the ESCRT-III complex. Independent of our work, Watanabe et al. (20) showed that the release of a different herpesvirus was susceptible to blockage by a prazole drug. They also used an HIV-1 Gag mutant bearing a disrupted PT/SAP motif (P7L-Gag) whose virus egress was independent of Tsg101 to demonstrate that the release of this virus mutant was still blocked. This indicates that prazole drugs in particular are effective in blocking the budding process.

While the prazole analogs block the release of lenti- and herpesviruses, the inhibition is manifested in different regions of the cell. The drugs block the release of HIV-1 at the outer cell membrane by preventing the pinching of virus particles from the membrane (3). In contrast, herpesviruses, which assemble in the nucleus, appear to be first blocked at the passage of the virus through the nuclear membrane. Because the prazole drugs form a covalent bond to Tsg101, this strongly suggests that the ESCRT proteins are important for the herpesvirus particles to be released from the nucleus of the cell where they are formed. This is consistent with a recent report by Arii et al. (18) that the ESCRT-III protein complex mediates herpesvirus movement across the nuclear membrane and regulates its integrity. The finding that the prazole drugs cause a significant drop in total infectious herpesviruses reported here can be explained by the trapping of immature particles in the nucleus. This prevents them from migrating into the cytoplasm to exchange enveloped membranes, which makes them infectious. Also, the accumulation of the dense material in the nucleus observed in the electron micrographs suggests that prazole drugs may interfere with normal particle assembly in addition to blocking the passage of the particles through the nuclear membrane.

The use of prazoles represents an exciting potential case of repurposing existing drugs to act as antiviral therapeutic agents. Currently, omeprazole is marketed as a prodrug for the treatment of acid reflux disease. Other prazole drugs are marketed for the treatment of acid reflux disease in China, India, and South Korea (Yi Li An, Adiza, and Noltec, respectively), indicating reasonable bioavailability and a known clinical safety profile. The prodrugs are acid activated into derivatives that form disulfide linkages with proton pumps (26, 61, 62). The prodrug, but not the charged sulfonamide derivative, can cross the plasma membrane barrier. The antiviral activity of tenatoprazole has been suggested to be the result of forming a covalent disulfide bond with Tsg101 (3). While the binding site for tenatoprazole is near the ubiquitin (Ub)-binding pocket and not the L-domain-binding site, biochemical and confocal imaging data independently demonstrated that tenatoprazole disrupts the binding of Tsg101 to the PT/SAP sequence (3). While the precise biochemical mechanism remains to be clarified, our FTS results support that it may be related to allosteric changes in Tsg101 after the drug forms its covalent linkage with Cys73. Previous reports did not detect off-target effects of the prazole drugs affecting Tsg101 metabolism inside cells (3). A possible exception is noted in a recent epidemiological study in the *American Journal of Gastroenterology* by C. V. Almario and colleagues (63). In these studies, there was a small correlation between severe acute respiratory syndrome coronavirus 2 (SARS-CoV-2) infections and patients taking commercially available prazole drugs, such as omeprazole, for acid reflux disease. However, this does not preclude the use of the prazole compounds described in this paper. The drugs used by the patients, such as omeprazole, have weak antiviral activity (Table 2). In contrast, ilaprazole has potent antiviral activity. At a dose of 10 mg of ilaprazole/day, plasma concentrations are around 2 μg h/ml, which is within the range needed for antiviral activity (64). The prazoles that we tested here also appear to be nontoxic to Vero, HeLa, and 293T cells at the concentrations used to inhibit the budding of herpesviruses and HIV-1. To improve the potency of the prazole drugs, we have synthesized 53 analogs of ilaprazole. Several of these appear to have stronger binding to Tsg101 detected by the FTS assay. We are now testing these analogs to see if they have more potent antiviral activity than ilaprazole.

A recent report highlighted the potential of prazole compounds to have a therapeutic effect on SARS-CoV-2 when combined with remdesivir (65). However, those authors did not definitively identify the mechanism of action of the prazoles and also concluded that the potency of the prazole compound used, omeprazole, is too low to reach therapeutic levels *in vivo*. A mechanism posed by those authors is that the prazoles lead to an increase in the lysosomal pH, which is the potential mechanism for lysosomotropic drugs such as chloroquine (66). In contrast to omeprazole, we hypothesize that ilaprazole may allow for therapeutic levels to be reached *in vivo*. In the case of ilaprazole, which is marketed in several Asian countries, as discussed above, our strong *in vitro* results lay the foundation for a potential fast track to broad-spectrum antiviral clinical testing, alone or in combination with other drugs, in these countries. We are currently working to determine if ilaprazole or our novel compounds have activity against SARS-CoV-2 with or in combination with remdesivir. This would further the potential broad-spectrum antiviral capacity of the prazole compounds described in this report.

## MATERIALS AND METHODS

### Viruses, plasmids, and cell lines.

Herpes simplex virus 1 (Kos strain), herpes simplex virus 2 (A/B-G), HIV plasmid pR9-HIV-1_Ba-L_ (Center For AIDS Research [CFAR] Lab), the pET-28b vector (Novagen-EMD Millipore), Rosetta2(DE3)/pLysS Escherichia coli competent cells (EMD Millipore), Vero cells, and 293T cell lines were used.

### Chemicals.

The prazole compounds rabeprazole, lansoprazole, omeprazole, ilaprazole, dexlansoprazole, tenatoprazole, and pantoprazole were obtained from SelleckChem. 2-[(4-Ethoxy-3-methylpridin-2-yl)methanesulfinyl]-1H-1,3-benzodiazole, 2-[(3,5-dimethylpyridin-2-yl)methanesulfinyl]-5-methoxy-1H-1,3-benzodiazole, and 4-methoxy-2-{[(5-methoxy-1H-1,3-benzodiazol-2-yl)sulfinyl]methyl}-3,5-dimethyl-1λ-pyridin-1-one were obtained from MolPort. Esomeprazole was obtained from Toronto Research Chemicals.

### Purification of Tsg101(1–145).

The N-terminally His_6_-tagged Tsg101 UEV domain (amino acids 1 to 145), called Tsg101-UEV, was encoded in a pET-28b vector (Novagen-EMD Millipore), which also included a thrombin protease cleavage site [His_6_-thrombin site-Tsg101(1–145)]. Tsg101-UEV was grown in LB broth with kanamycin (30 μg/ml) in Rosetta2(DE3)/pLysS E. coli competent cells (EMD Millipore) and induced with 1 mM isopropyl-β-d-thiogalactopyranoside (IPTG) at room temperature for 3 h. Bacteria were collected by centrifugation at 4,000 rpm for 10 min at 4°C. Bacteria were suspended in 50 ml binding buffer (20 mM Tris-HCl [pH 7.9], 0.5 M NaCl, 5 mM imidazole) containing 1 mM phenylmethylsulfonyl fluoride (PMSF), 0.1% NP-40, and a protease inhibitor cocktail tablet (Roche) and sonicated for 3.5 min on ice. The sonicate was spun at 9,000 rpm for 1 h at 4°C in a Sorvall centrifuge. The supernatant was collected and passed through a 1.5-ml Ni-nitrilotriacetic acid (NTA) agarose column. The column was washed with wash buffer containing 20 mM Tris-HCl (pH 7.9), 0.5 M NaCl, and 30 mM imidazole. The column was then equilibrated with tobacco etch virus (TEV) cleavage buffer followed by 50 U of thrombin in the same buffer (Novagene). The column flow was stopped, and the mixture was incubated at room temperature overnight. The cleaved protein was eluted with wash buffer, and the protein was dialyzed in D-tube Dialyzer Maxi, with a molecular weight cutoff (MWCO) of 12 to 14 kDa (Novagene), overnight against buffer containing 0.15 M NaCl and 0.1 M HEPES (pH 7.5). The protein was concentrated in a MicroSep advanced centrifugal device, with 12- to 14-kDa exclusion (Pall), for 1 h at 1,300 rpm. The protein concentration was determined with a NanoDrop spectrophotometer at 280 nm. When the His tag was not removed, the protein was eluted from the Ni-NTA column with a solution containing 20 mM Tris-HCl (pH 7.9), 0.5 M NaCl, and 1 M imidazole. The protein was evaluated by SDS-PAGE for purity.

### Fluorescence thermal shift screening to identify small molecules binding to Tsg101-UEV.

The fluorescence thermal shift (FTS) assay uses the thermal shift elicited by the effect of small-molecule binding on protein stability. The FTS assay monitors protein thermal denaturation using the environment-sensitive dye SYPRO orange, which fluoresces when bound to hydrophobic surfaces, taking advantage of the changes in hydrophobic surface exposure in protein denaturation. The discovery of small molecules binding to the target protein utilizes the observation that ligand binding affects protein thermal stability and therefore can be detected through a shift in the protein’s thermal denaturation (melting) temperature (*T_m_*). We have employed the FTS assay to reveal changes in the thermodynamic properties of Tsg101 elicited by its interaction with a small molecule. The recombinant Tsg101 fragment (amino acids 1 to 145), prepared as described above (but without a label), has a thermal unfolding profile suitable for using the FTS assay as a primary screening assay for high-throughput screening (HTS). A fluorescence dye, SYPRO orange (Invitrogen), was used for assay detection. The dye is excited at 473 nm and has a fluorescence emission at 610 nm. The dye binds to hydrophobic regions of a protein that are normally buried in a native protein structure. When a protein is unfolded, the dye interacts with exposed hydrophobic surfaces, and the fluorescence intensity increases significantly over that observed in aqueous solution. The Tsg101 fragment was premixed at a concentration of 2 μM with a 5× concentration of SYPRO orange in HEPES buffer (100 mM HEPES, 150 mM NaCl [pH 7.5]). Next, 10 μl of the protein-dye mix was added to an assay plate, and 10 to 50 nl of the compound, equal to 10 to 50 μM, was added with an Echo550 acoustic-transfer robot (Labcyte, CA). The plate was shaken to ensure proper mixing, sealed with an optical seal, and then centrifuged. The thermal scan was performed from 20°C to 90°C with a temperature ramp rate of 0.5°C/min. Fluorescence was detected on a CFX384 real-time PCR machine (Bio-Rad Laboratories). Comparison of the thermal denaturation profiles for Tsg101-UEV in the presence and absence of tenatoprazole and other prazoles revealed destabilization of the native protein structure, indicating that the compound interacted with Tsg101-UEV.

### Herpesvirus infection of Vero cells.

Vero cells (0.8 × 10^6^ cells/well of a 6-well plate) were infected with HSV-1 or HSV-2 at an MOI of 0.1 PFU/cell in Dulbecco’s modified Eagle’s medium (DMEM) with 1% serum for 2 h in a CO_2_ incubator at 37°C. In one experiment looking at the effect of tenatoprazole on HSV-2 release from cells at 24 and 48 h, an MOI of 3 PFU/cell was used. The cell supernatants were aspirated and replaced with 1 ml (24 h) or 2 ml (48 h and 72 h) of DMEM with 1% serum with dimethyl sulfoxide (DMSO) or different concentrations of the drug (tenatoprazole, ilaprazole, or analogs) dissolved in DMSO. After 24 or 48 h of incubation, the cell supernatant was collected and frozen at −80°C. The virus titer in the cell medium fraction was determined by standard plaque assays using 10-fold serial dilutions of cell supernatants of Vero cells incubated for 48 h, after which cells were fixed and stained to count the plaques (22). For the determination of total virus (extracellular and cytoplasmic), virus-infected cells were incubated for 24 h with and without drug presence, and the plate of cells was then subjected to 3 cycles of freeze-thawing (−80°C/37°C) for 30 min each prior to collecting the supernatant after centrifugation for measurement of the total virus titer. The virus titer was measured by standard plaque assays as described above. For analyzing the effect of benserazide (K21) at different concentrations on the release of HSV-1 from Vero cells, experiments were repeated 4 times each and did not appear to affect the release of virus from cells. In separate experiments, uninfected Vero cells were carried for 3 weeks in culture in the presence or absence of drugs (replaced every third day) and found to exhibit the same growth rate detected with a light microscope.

### HIV-1 transfection of 293T cells.

293T cells (American Type Culture Collection) were grown in a 24-well, clear, flat-bottom, tissue culture-treated multiwell cell culture plate using Dulbecco’s modified Eagle’s medium (Cellgro) containing fetal bovine serum (10%), 100 U/ml penicillin, 100 μg/ml streptomycin, and 292 μg/ml l-glutamine (Cellgro). Cells were grown to 60 to 70% confluence at 37°C with 5% CO_2_ prior to the addition of drug treatment. Culture medium was aspirated and replaced with medium containing the drug compound 7 h prior to transfection of the plasmid encoding the HIV-1 genome. Transfection was done using polyethyleneimine (PEI) reagent (Polysciences). For the production of virus particles, cells were transfected with the pR9-HIV-1_Ba-L_ plasmid. After 24 h and 48 h, tissue culture medium was collected and passed through a 0.45-μm filter. Virus released from cells was quantified by medium-associated p24 determined using a fluorescently tagged CA-targeting antibody (PerkinElmer) and equivalent amounts of p24 as standards.

### Drug potency and cell toxicity.

EC_50_ calculations were performed by using AAT Bioquest’s EC_50_ calculator. Cell toxicity at different concentrations of drugs as indicated was determined using the WST-1 cell proliferation reagent (Roche Diagnostics) or the cellular 96 AQueous One viability reagent according to the manufacturer’s instructions. For 293T cells, the concentration of DMSO was 0.2% or lower, and assays were carried out with DMEM with 10% serum. Cell toxicity experiments were repeated twice.

### Transmission electron microscopy.

Vero cells on glass coverslips were infected with HSV-2 at an MOI of 0.1 for 2 h. Next, 105 μM tenatoprazole or 18 μM ilaprazole was added, and cells were incubated for 24 h. Tissue samples were fixed in 0.1 M sodium cacodylate buffer (pH 7.3) containing 2% paraformaldehyde and 2.5% glutaraldehyde and postfixed with 2% osmium tetroxide in an unbuffered aqueous solution. The samples were rinsed with distilled water, *en bloc* stained with 3% uranyl acetate, rinsed with distilled water, dehydrated in ascending grades of ethanol, transitioned with propylene oxide, embedded in the resin mixture of the Embed 812 kit, and cured in a 60°C oven. Samples were sectioned on a Leica Ultracut UC6 ultramicrotome. One-micrometer-thick sections were collected and stained with toluidine blue O, and 70-nm sections were collected on 200-mesh copper grids; thin sections were stained with uranyl acetate and Reynolds’ lead citrate. Transmission electron microscopy (TEM) was performed on an FEI Tecnai Spirit G2 instrument.

## References

[1] Gottlinger HG, Dorfman T, Sodroski JG, Haseltine WA. 1991. Effect of mutations affecting the p6 gag protein on human immunodeficiency virus particle release. Proc Natl Acad Sci U S A 88:3195–3199. 10.1073/pnas.88.8.3195. 2014240 PMC51412

[2] Pincetic A, Medina G, Carter C, Leis J. 2008. Avian sarcoma virus and human immunodeficiency virus, type 1 use different subsets of ESCRT proteins to facilitate the budding process. J Biol Chem 283:29822–29830. 10.1074/jbc.M804157200. 18723511 PMC2573067

[3] Strickland M, Ehrlich LS, Watanabe S, Khan M, Strub MP, Luan CH, Powell MD, Leis J, Tjandra N, Carter CA. 2017. Tsg101 chaperone function revealed by HIV-1 assembly inhibitors. Nat Commun 8:1391. 10.1038/s41467-017-01426-2. 29123089 PMC5680296

[4] Wills JW, Cameron CE, Wilson CB, Xiang Y, Bennett RP, Leis J. 1994. An assembly domain of the Rous sarcoma virus Gag protein required late in budding. J Virol 68:6605–6618. 10.1128/JVI.68.10.6605-6618.1994. 8083996 PMC237081

[5] Xiang Y, Cameron CE, Wills JW, Leis J. 1996. Fine mapping and characterization of the Rous sarcoma virus Pr76gag late assembly domain. J Virol 70:5695–5700. 10.1128/JVI.70.8.5695-5700.1996. 8764091 PMC190537

[6] Medina G, Pincetic A, Ehrlich LS, Zhang Y, Tang Y, Leis J, Carter CA. 2008. Tsg101 can replace Nedd4 function in ASV Gag release but not membrane targeting. Virology 377:30–38. 10.1016/j.virol.2008.04.024. 18555885 PMC2528022

[7] Taylor GM, Hanson PI, Kielian M. 2007. Ubiquitin depletion and dominant-negative VPS4 inhibit rhabdovirus budding without affecting alphavirus budding. J Virol 81:13631–13639. 10.1128/JVI.01688-07. 17913808 PMC2168838

[8] Medina G, Zhang Y, Tang Y, Gottwein E, Vana ML, Bouamr F, Leis J, Carter CA. 2005. The functionally exchangeable L domains in RSV and HIV‐1 Gag direct particle release through pathways linked by Tsg101. Traffic 6:880–894. 10.1111/j.1600-0854.2005.00323.x. 16138902 PMC2692930

[9] VerPlank L, Bouamr F, LaGrassa TJ, Agresta B, Kikonyogo A, Leis J, Carter CA. 2001. Tsg101, a homologue of ubiquitin-conjugating (E2) enzymes, binds the L domain in HIV type 1 Pr55Gag. Proc Natl Acad Sci U S A 98:7724–7729. 10.1073/pnas.131059198. 11427703 PMC35409

[10] Chung HY, Morita E, von Schwedler U, Muller B, Krausslich HG, Sundquist WI. 2008. NEDD4L overexpression rescues the release and infectivity of human immunodeficiency virus type 1 constructs lacking PTAP and YPXL late domains. J Virol 82:4884–4897. 10.1128/JVI.02667-07. 18321968 PMC2346761

[11] Fujii K, Munshi UM, Ablan SD, Demirov DG, Soheilian F, Nagashima K, Stephen AG, Fisher RJ, Freed EO. 2009. Functional role of Alix in HIV-1 replication. Virology 391:284–292. 10.1016/j.virol.2009.06.016. 19596386 PMC2744943

[12] Garrus JE, von Schwedler UK, Pornillos OW, Morham SG, Zavitz KH, Wang HE, Wettstein DA, Stray KM, Cote M, Rich RL, Myszka DG, Sundquist WI. 2001. Tsg101 and the vacuolar protein sorting pathway are essential for HIV-1 budding. Cell 107:55–65. 10.1016/s0092-8674(01)00506-2. 11595185

[13] Goff A, Ehrlich LS, Cohen SN, Carter CA. 2003. Tsg101 control of human immunodeficiency virus type 1 Gag trafficking and release. J Virol 77:9173–9182. 10.1128/jvi.77.17.9173-9182.2003. 12915533 PMC187429

[14] Martin-Serrano J, Yarovoy A, Perez-Caballero D, Bieniasz PD. 2003. Divergent retroviral late-budding domains recruit vacuolar protein sorting factors by using alternative adaptor proteins. Proc Natl Acad Sci U S A 100:12414–12419. 10.1073/pnas.2133846100. 14519844 PMC218772

[15] Pornillos O, Alam SL, Rich RL, Myszka DG, Davis DR, Sundquist WI. 2002. Structure and functional interactions of the Tsg101 UEV domain. EMBO J 21:2397–2406. 10.1093/emboj/21.10.2397. 12006492 PMC125378

[16] von Schwedler UK, Stuchell M, Muller B, Ward DM, Chung HY, Morita E, Wang HE, Davis T, He GP, Cimbora DM, Scott A, Krausslich HG, Kaplan J, Morham SG, Sundquist WI. 2003. The protein network of HIV budding. Cell 114:701–713. 10.1016/s0092-8674(03)00714-1. 14505570

[17] Strack B, Calistri A, Craig S, Popova E, Göttlinger HG. 2003. AIP1/ALIX is a binding partner for HIV-1 p6 and EIAV p9 functioning in virus budding. Cell 114:689–699. 10.1016/s0092-8674(03)00653-6. 14505569

[18] Arii J, Watanabe M, Maeda F, Tokai-Nishizumi N, Chihara T, Miura M, Maruzuru Y, Koyanagi N, Kato A, Kawaguchi Y. 2018. ESCRT-III mediates budding across the inner nuclear membrane and regulates its integrity. Nat Commun 9:3379. 10.1038/s41467-018-05889-9. 30139939 PMC6107581

[19] Lee CP, Liu PT, Kung HN, Su MT, Chua HH, Chang YH, Chang CW, Tsai CH, Liu FT, Chen MR. 2012. The ESCRT machinery is recruited by the viral BFRF1 protein to the nucleus-associated membrane for the maturation of Epstein-Barr virus. PLoS Pathog 8:e1002904. 10.1371/journal.ppat.1002904. 22969426 PMC3435242

[20] Watanabe SM, Ehrlich LS, Strickland M, Li X, Soloveva V, Goff AJ, Stauft CB, Bhaduri-McIntosh S, Tjandra N, Carter C. 2020. Selective targeting of virus replication by proton pump inhibitors. Sci Rep 10:4003. 10.1038/s41598-020-60544-y. 32132561 PMC7055211

[21] Seo EJ, Leis J. 2012. Budding of enveloped viruses: interferon-induced ISG15—antivirus mechanisms targeting the release process. Adv Virol 2012:532723. 10.1155/2012/532723. 22666250 PMC3362814

[22] Kuang Z, Seo EJ, Leis J. 2011. Mechanism of inhibition of retrovirus release from cells by interferon-induced gene ISG15. J Virol 85:7153–7161. 10.1128/JVI.02610-10. 21543490 PMC3126601

[23] Pincetic A, Leis J. 2009. The mechanism of budding of retroviruses from cell membranes. Adv Virol 2009:6239699. 10.1155/2009/623969. PMC2768365 19865606

[24] Pincetic A, Kuang Z, Seo EJ, Leis J. 2010. The interferon-induced gene ISG15 blocks retrovirus release from cells late in the budding process. J Virol 84:4725–4736. 10.1128/JVI.02478-09. 20164219 PMC2863725

[25] Bashirova AA, Bleiber G, Qi Y, Hutcheson H, Yamashita T, Johnson RC, Cheng J, Alter G, Goedert JJ, Buchbinder S, Hoots K, Vlahov D, May M, Maldarelli F, Jacobson L, O’Brien SJ, Telenti A, Carrington M. 2006. Consistent effects of TSG101 genetic variability on multiple outcomes of exposure to human immunodeficiency virus type 1. J Virol 80:6757–6763. 10.1128/jvi.00094-06. 16809281 PMC1489017

[26] Shin JM, Kim N. 2013. Pharmacokinetics and pharmacodynamics of the proton pump inhibitors. J Neurogastroenterol Motil 19:25–35. 10.5056/jnm.2013.19.1.25. 23350044 PMC3548122

[27] Luan C-H, Light SH, Dunne SF, Anderson WF. 2014. Ligand screening using fluorescence thermal shift analysis (FTS). Methods Mol Biol 1140:263–289. 10.1007/978-1-4939-0354-2_20. 24590724

[28] Pantoliano MW, Petrella EC, Kwasnoski JD, Lobanov VS, Myslik J, Graf E, Carver T, Asel E, Springer BA, Lane P, Salemme FR. 2001. High-density miniaturized thermal shift assays as a general strategy for drug discovery. J Biomol Screen 6:429–440. 10.1177/108705710100600609. 11788061

[29] Lee SK, Longnecker R. 1997. The Epstein-Barr virus glycoprotein 110 carboxy-terminal tail domain is essential for lytic virus replication. J Virol 71:4092–4097. 10.1128/JVI.71.5.4092-4097.1997. 9094688 PMC191563

[30] Pawliczek T, Crump CM. 2009. Herpes simplex virus type 1 production requires a functional ESCRT-III complex but is independent of TSG101 and ALIX expression. J Virol 83:11254–11264. 10.1128/JVI.00574-09. 19692479 PMC2772754

[31] Calistri A, Sette P, Salata C, Cancellotti E, Forghieri C, Comin A, Göttlinger H, Campadelli-Fiume G, Palù G, Parolin C. 2007. Intracellular trafficking and maturation of herpes simplex virus type 1 gB and virus egress require functional biogenesis of multivesicular bodies. J Virol 81:11468–11478. 10.1128/JVI.01364-07. 17686835 PMC2045546

[32] Calistri A, Munegato D, Toffoletto M, Celestino M, Franchin E, Comin A, Sartori E, Salata C, Parolin C, Palu G. 2015. Functional interaction between the ESCRT‐I component TSG101 and the HSV‐1 tegument ubiquitin specific protease. J Cell Physiol 230:1794–1806. 10.1002/jcp.24890. 25510868

[33] Crump CM, Yates C, Minson T. 2007. Herpes simplex virus type 1 cytoplasmic envelopment requires functional Vps4. J Virol 81:7380–7387. 10.1128/JVI.00222-07. 17507493 PMC1933334

[34] Tandon R, AuCoin DP, Mocarski ES. 2009. Human cytomegalovirus exploits ESCRT machinery in the process of virion maturation. J Virol 83:10797–10807. 10.1128/JVI.01093-09. 19640981 PMC2753131

[35] Perez M, Craven RC, de la Torre JC. 2003. The small RING finger protein Z drives arenavirus budding: implications for antiviral strategies. Proc Natl Acad Sci U S A 100:12978–12983. 10.1073/pnas.2133782100. 14563923 PMC240730

[36] Urata S, Noda T, Kawaoka Y, Yokosawa H, Yasuda J. 2006. Cellular factors required for Lassa virus budding. J Virol 80:4191–4195. 10.1128/JVI.80.8.4191-4195.2006. 16571837 PMC1440458

[37] Ariumi Y, Kuroki M, Maki M, Ikeda M, Dansako H, Wakita T, Kato N. 2011. The ESCRT system is required for hepatitis C virus production. PLoS One 6:e14517. 10.1371/journal.pone.0014517. 21264300 PMC3019154

[38] Corless L, Crump CM, Griffin SD, Harris M. 2010. Vps4 and the ESCRT-III complex are required for the release of infectious hepatitis C virus particles. J Gen Virol 91:362–372. 10.1099/vir.0.017285-0. 19828764 PMC7615705

[39] Han Z, Lu J, Liu Y, Davis B, Lee MS, Olson MA, Ruthel G, Freedman BD, Schnell MJ, Wrobel JE, Reitz AB, Harty RN. 2014. Small-molecule probes targeting the viral PPxY-host Nedd4 interface block egress of a broad range of RNA viruses. J Virol 88:7294–7306. 10.1128/JVI.00591-14. 24741084 PMC4054416

[40] Harty RN, Brown ME, Wang G, Huibregtse J, Hayes FP. 2000. A PPxY motif within the VP40 protein of Ebola virus interacts physically and functionally with a ubiquitin ligase: implications for filovirus budding. Proc Natl Acad Sci U S A 97:13871–13876. 10.1073/pnas.250277297. 11095724 PMC17668

[41] Lu J, Han Z, Liu Y, Liu W, Lee MS, Olson MA, Ruthel G, Freedman BD, Harty RN. 2014. A host-oriented inhibitor of Junin Argentine hemorrhagic fever virus egress. J Virol 88:4736–4743. 10.1128/JVI.03757-13. 24522922 PMC3993831

[42] Madara JJ, Han Z, Ruthel G, Freedman BD, Harty RN. 2015. The multifunctional Ebola virus VP40 matrix protein is a promising therapeutic target. Future Virol 10:537–546. 10.2217/fvl.15.6. 26120351 PMC4480923

[43] Martin-Serrano J, Zang T, Bieniasz PD. 2001. HIV-1 and Ebola virus encode small peptide motifs that recruit Tsg101 to sites of particle assembly to facilitate egress. Nat Med 7:1313–1319. 10.1038/nm1201-1313. 11726971

[44] Silvestri LS, Ruthel G, Kallstrom G, Warfield KL, Swenson DL, Nelle T, Iversen PL, Bavari S, Aman MJ. 2007. Involvement of vacuolar protein sorting pathway in Ebola virus release independent of TSG101 interaction. J Infect Dis 196(Suppl 2):S264–S270. 10.1086/520610. 17940959

[45] Timmins J, Schoehn G, Ricard-Blum S, Scianimanico S, Vernet T, Ruigrok RW, Weissenhorn W. 2003. Ebola virus matrix protein VP40 interaction with human cellular factors Tsg101 and Nedd4. J Mol Biol 326:493–502. 10.1016/s0022-2836(02)01406-7. 12559917

[46] Urata S, Noda T, Kawaoka Y, Morikawa S, Yokosawa H, Yasuda J. 2007. Interaction of Tsg101 with Marburg virus VP40 depends on the PPPY motif, but not the PT/SAP motif as in the case of Ebola virus, and Tsg101 plays a critical role in the budding of Marburg virus-like particles induced by VP40, NP, and GP. J Virol 81:4895–4899. 10.1128/JVI.02829-06. 17301151 PMC1900181

[47] Lambert C, Döring T, Prange R. 2007. Hepatitis B virus maturation is sensitive to functional inhibition of ESCRT-III, Vps4, and γ2-adaptin. J Virol 81:9050–9060. 10.1128/JVI.00479-07. 17553870 PMC1951427

[48] Li M, Schmitt PT, Li Z, McCrory TS, He B, Schmitt AP. 2009. Mumps virus matrix, fusion, and nucleocapsid proteins cooperate for efficient production of virus-like particles. J Virol 83:7261–7272. 10.1128/JVI.00421-09. 19439476 PMC2704775

[49] Schmitt AP, Leser GP, Morita E, Sundquist WI, Lamb RA. 2005. Evidence for a new viral late-domain core sequence, FPIV, necessary for budding of a paramyxovirus. J Virol 79:2988–2997. 10.1128/JVI.79.5.2988-2997.2005. 15709019 PMC548478

[50] Schmitt AP, Leser GP, Waning DL, Lamb RA. 2002. Requirements for budding of paramyxovirus simian virus 5 virus-like particles. J Virol 76:3952–3964. 10.1128/jvi.76.8.3952-3964.2002. 11907235 PMC136107

[51] Irie T, Harty RN. 2005. L-domain flanking sequences are important for host interactions and efficient budding of vesicular stomatitis virus recombinants. J Virol 79:12617–12622. 10.1128/JVI.79.20.12617-12622.2005. 16188963 PMC1235845

[52] Wirblich C, Tan GS, Papaneri A, Godlewski PJ, Orenstein JM, Harty RN, Schnell MJ. 2008. PPEY motif within the rabies virus (RV) matrix protein is essential for efficient virion release and RV pathogenicity. J Virol 82:9730–9738. 10.1128/JVI.00889-08. 18667490 PMC2546990

[53] Gordon CJ, Tchesnokov EP, Feng JY, Porter DP, Gotte M. 2020. The antiviral compound remdesivir potently inhibits RNA-dependent RNA polymerase from Middle East respiratory syndrome coronavirus. J Biol Chem 295:4773–4779. 10.1074/jbc.AC120.013056. 32094225 PMC7152756

[54] Arguello MD, Hiscott J. 2007. Ub surprised: viral ovarian tumor domain proteases remove ubiquitin and ISG15 conjugates. Cell Host Microbe 2:367–369. 10.1016/j.chom.2007.11.005. 18078688

[55] Frias-Staheli N, Giannakopoulos NV, Kikkert M, Taylor SL, Bridgen A, Paragas J, Richt JA, Rowland RR, Schmaljohn CS, Lenschow DJ, Snijder EJ, García-Sastre A, Virgin HW, IV. 2007. Ovarian tumor domain-containing viral proteases evade ubiquitin- and ISG15-dependent innate immune responses. Cell Host Microbe 2:404–416. 10.1016/j.chom.2007.09.014. 18078692 PMC2184509

[56] Harty RN, Pitha PM, Okumura A. 2009. Antiviral activity of innate immune protein ISG15. J Innate Immun 1:397–404. 10.1159/000226245. 19680460 PMC2725329

[57] Vana ML, Tang Y, Chen A, Medina G, Carter C, Leis J. 2004. Role of Nedd4 and ubiquitination of Rous sarcoma virus Gag in budding of virus-like particles from cells. J Virol 78:13943–13953. 10.1128/JVI.78.24.13943-13953.2004. 15564502 PMC533940

[58] Yuan W, Aramini JM, Montelione GT, Krug RM. 2002. Structural basis for ubiquitin-like ISG 15 protein binding to the NS1 protein of influenza B virus: a protein-protein interaction function that is not shared by the corresponding N-terminal domain of the NS1 protein of influenza A virus. Virology 304:291–301. 10.1006/viro.2002.1663. 12504570

[59] Yuan W, Krug RM. 2001. Influenza B virus NS1 protein inhibits conjugation of the interferon (IFN)-induced ubiquitin-like ISG15 protein. EMBO J 20:362–371. 10.1093/emboj/20.3.362. 11157743 PMC133459

[60] Usami Y, Popov S, Popova E, Gottlinger HG. 2008. Efficient and specific rescue of human immunodeficiency virus type 1 budding defects by a Nedd4-like ubiquitin ligase. J Virol 82:4898–4907. 10.1128/JVI.02675-07. 18321969 PMC2346742

[61] Kakinoki B, Ono C, Yamazaki N, Chikamatsu N, Wakatsuki D, Uchiyama K, Morinaka Y. 1999. General pharmacological properties of the new proton pump inhibitor (+/−)-5-methoxy-2-(((4-methoxy-3,5-dimethylpyrid-2-yl)methyl)sulfinyl)-1H-imidazo(4,5-b)pryidine. Methods Find Exp Clin Pharmacol 21:179–187. 10.1358/mf.1999.21.3.534827. 10389120

[62] Shin JM, Sachs G. 2002. Restoration of acid secretion following treatment with proton pump inhibitors. Gastroenterology 123:1588–1597. 10.1053/gast.2002.36593. 12404233

[63] Almario CV, Chey WD, Spiegel BMR. 2020. Increased risk of COVID-19 among users of proton pump inhibitors. Am J Gastroenterol 115:1707–1715. 10.14309/ajg.0000000000000798. 32852340 PMC7473791

[64] de Bortoli N, Martinucci I, Giacchino M, Blandizzi C, Marchi S, Savarino V, Savarino E. 2013. The pharmacokinetics of ilaprazole for gastro-esophageal reflux treatment. Expert Opin Drug Metab Toxicol 9:1361–1369. 10.1517/17425255.2013.813018. 23802731

[65] Bojkova D, McGreig JE, McLaughlin K-M, Masterson SG, Widera M, Kraehling V, Ciesek S, Wass MN, Michaelis M, Cinatl JN. 2020. SARS-CoV-2 and SARS-CoV differ in their cell tropism and drug sensitivity profiles. bioRxiv 10.1101/2020.04.03.024257.

[66] Al-Bari MAA. 2017. Targeting endosomal acidification by chloroquine analogs as a promising strategy for the treatment of emerging viral diseases. Pharmacol Res Perspect 5:e00293. 10.1002/prp2.293. 28596841 PMC5461643

[67] Carlton JG, Martin-Serrano J. 2007. Parallels between cytokinesis and retroviral budding: a role for the ESCRT machinery. Science 316:1908–1912. 10.1126/science.1143422. 17556548

